# A chemically optimized, GalNAc-conjugated siRNA targeting HSD17B13 demonstrates efficacy in a human 3D organoid model of MASH, showcasing an alternative to animal testing

**DOI:** 10.1371/journal.pone.0354751

**Published:** 2026-08-14

**Authors:** Qin Meng, Xujia Wang, Yuxuan Zhang, Sudeep Pushpakom, Jun Yang, Hui Feng, Mu Wang

**Affiliations:** 1 Academy of Pharmacy, Xi’an Jiaotong-Liverpool University, Suzhou, China; 2 Department of Analytical Science, Shanghai Junshi Biosciences Co., Ltd., Shanghai, China; 3 Institute of Systems, Molecular & Integrative Biology, Faculty of Health and Life Sciences, University of Liverpool, Liverpool, United Kingdom; 4 Reasearch & Development Department, Shanghai Anlingke Biopharmaceutical Co., Ltd., Shanghai, China; King Saud University / Zagazig University, EGYPT

## Abstract

**Introduction:**

Animal models have limited ability to predict human metabolically dysfunction-associated steatohepatitis (MASH), creating a major obstacle in drug development. To address this challenge, we introduce an integrated drug discovery approach that combines rational small-interfering RNA (siRNA) design and chemical modifications to improve stability, validated in a complex human 3D NAC-organoid MASH model.

**Methods:**

Using this system, we created new GalNAc-conjugated siRNAs targeting the 17β-hydroxysteroid dehydrogenase 13 (HSD17B13). The lead candidates were evaluated for stability in human serum and liver microsomes, off-target risks, and efficacy in the 3D organoid model that mimics key disease features such as steatosis and fibrosis.

**Results:**

Our main candidate, si-R5-42, demonstrated greater stability in human serum and liver microsomes, as well as fewer off-target risks. Most importantly, in the 3D organoid model, si-R5-42 successfully reduced disease markers. Its ability to lower hepatic steatosis and fibrogenesis was comparable to that of the clinical-phase candidate ARO-HSD.

**Conclusion:**

This research provides a promising therapeutic candidate and a solid, human-relevant preclinical siRNA testing framework, reducing dependence on animal models.

## Introduction

Metabolically-dysfunction-associated steatohepatitis (MASH), a progressive form of chronic liver disease, represents a significant and growing global health burden with no universally approved pharmacotherapy [1]. Its pathogenesis involves a complex interplay of steatosis, inflammation, and fibrosis, ultimately leading to cirrhosis and hepatocellular carcinoma (HCC) [2,3]. The prevalence of MASH is rising in tandem with obesity and metabolic syndrome, underscoring the urgent need for effective treatments [4,5]. While the recent accelerated approval of Resmetirom marks a milestone, the therapeutic landscape remains sparse, underscoring the challenges in targeting this multifaceted disease [6].

The 17β-hydroxysteroid dehydrogenase 13 (HSD17B13) gene has emerged as a promising therapeutic target [7]. Human genetic studies robustly associate loss-of-function variants in HSD17B13 with a reduced risk of chronic liver disease, positioning it as a high-confidence target for MASH intervention [8–11]. This validation has spurred the development of HSD17B13-targeted therapies, most notably ARO-HSD (Arrowhead Pharmaceuticals), a GalNAc-conjugated small-interfering RNA (siRNA) currently in Phase II clinical trials [11,12]. Early clinical data showing improved liver enzyme levels and fat fraction in subjects with suspected MASH confirm the therapeutic promise of silencing HSD17B13. Early clinical data showing improved liver enzyme levels, fat fraction, and stiffness in subjects with suspected MASH confirm the therapeutic promise of silencing HSD17B13 [12]. The HSD17B13 siRNA is expected to provide a breakthrough therapeutic option for MASH [9,13].

However, a critical bottleneck persists in the preclinical evaluation of novel MASH therapeutics, including siRNAs: the limited predictive value of existing animal models. Murine models of MASH often fail to fully recapitulate the human disease pathophysiology, particularly the progressive fibrotic response, leading to a high attrition rate of drug candidates in clinical trials [14,15]. This species-specific discrepancy necessitates the development of more human-relevant, predictive preclinical models to better prioritize candidates most likely to succeed in the clinic.

To address this translational gap, we established a human multicellular three-dimensional (3D) NAC-organoid model of MASH. This model, composed of primary human hepatocytes, hepatic stellate cells, liver sinusoidal endothelial cells, and Kupffer cells, mimics the key pathological features of human MASH — including steatosis, inflammation, and fibrosis—within a physiologically relevant tissue context [16]. We posited that this system would provide a superior platform for the functional validation of HSD17B13-targeting siRNAs, offering a bridge between conventional cell lines and the clinical reality.

Herein, we report an integrated strategy for the development of a novel siRNA therapeutic targeting HSD17B13. We utilized the OriSiRNA platform(Shanghai Junshi Biosciences) for rational design, employed strategic chemical modifications to enhance stability and minimize off-target effects, and conjugated our lead candidates with GalNAc for hepatocyte-specific delivery. Most importantly, we leveraged our 3D MASH organoid model as a rigorous, human-relevant testbed to evaluate the therapeutic efficacy of our optimized siRNAs, directly benchmarking them against the clinical-phase candidate ARO-HSD. This comprehensive approach not only identifies a promising therapeutic candidate but also establishes a generalizable pipeline for the development of siRNA-based medicines, reducing the reliance on poorly predictive animal models.

## Materials and methods

### siRNA synthesis and design

All initial siRNA sequences targeting human HSD17B13 mRNA were designed using the OriSiRNA platform. A total of 121 candidate sequences were selected based on the OriSiRNA scoring algorithm, patent landscape analysis, and literature review. Unmodified and chemically modified siRNA molecules, including the positive control ARO-HSD (sequence derived from Alnylam Pharmaceuticals patents), were synthesized by GenScript (Nanjing, China) and BioSyntech (Suzhou, China). The GalNAc conjugation was performed at the 3’-end of the sense strand of the final lead candidates.

### Cell culture and reporter gene assay (RGA)

HEK-293, Huh-7, and HepG2 cell lines were obtained from Shanghai Junshi Biosciences. HEK293-psiCheck-HSD17B13 reporter cells, which stably express a construct containing the full-length human HSD17B13 sequence inserted into the 3’ UTR of Renilla luciferase with Firefly luciferase as an internal control, were used for initial high-throughput screening.

HEK-293, Huh-7, and HepG2 cells were maintained in DMEM (Gibco) medium supplemented with 10% fetal bovine serum (FBS, Gibco) and 1% penicillin-streptomycin (Gibco) at 37°C in a 5% CO_2_ atmosphere. For the assay, each type of cell was seeded in 96-well white plates (Absin) at a density of 2 × 10⁴ cells per well, separately. Cells were then transfected with psiCheck-HSD17B13 reporter gene plasmid complex formulated by mixing equal volumes of Lipofectamine 3000 (Thermo Fisher Scientific) and dual reporter gene plasmid (psiCheck-2-HSD17B13) in Opti-MEM (Gibco). After 24 hours, cells were transfected with siRNA complexes formulated by mixing equal volumes of Lipofectamine RNAiMAX (Thermo Fisher Scientific) and siRNA (at various concentrations) in Opti-MEM (Gibco) medium. The positive control, ARO-HSD, was included in every assay plate for benchmark comparison. After 48 hours, luciferase activity was measured using the Dual-Luciferase Reporter Assay System (Promega) on a TECAN M1000 plate reader. Renilla luciferase signal was normalized to the Firefly luciferase signal for each well.

### Species cross-reactivity assay

To evaluate species specificity, HEK-293 cells were transiently co-transfected with plasmids expressing the *Renilla* luciferase gene fused to the HSD17B13 3’ UTR from human, cynomolgus monkey, or rhesus monkey, along with a *Firefly* luciferase control plasmid, using Lipofectamine 3000 (Thermo Fisher Scientific). Four hours post-transfection, siRNA transfection complexes were added as described above. Luciferase activities were measured after 48 hours, and the normalized *Renilla*/*Firefly* ratio was used to determine knockdown efficiency across species.

### HSD17B13 siRNA stability study

The metabolic stability of lead siRNA candidates was assessed in pooled human serum (GEMINI, 100512) and human liver microsomes (Corning, 452117). siRNA was spiked into the matrix to a final concentration of 3 μg/mL and incubated at 37°C. Aliquots were collected at 0, 1, 2, 4, 24, and 48 hours. Reactions were terminated by heating at 95°C for 5 minutes. The intact siRNA remaining was quantified using a stem-loop reverse transcription followed by real-time fluorescence quantitative polymerase chain reaction (qPCR), as described in the illustration. The percentage of remaining siRNA was calculated relative to the time-zero concentration. Inclisiran, an FDA-approved siRNA, was used as a reference control for stability.

### RNA sequencing and differential expression analysis

Huh-7 or HepG2 cells stably overexpressing HSD17B13 (Huh7-HSD/HepG2-HSD) were transfected with lead siRNA candidates or a non-targeting control siRNA (n = 5 per group). Total RNA was extracted 48 hours post-transfection. RNA sequences were analyzed on the DNBSEQ platform (BGI) with 50 bp single-end reads. Raw sequencing data were quality-controlled with FastQC. Differential gene expression analysis was performed using DESeq2 in R. Genes with an adjusted p-value < 0.05 and an absolute log2 fold-change > 1 were considered significant. Potential off-target effects were rigorously assessed by analyzing the number and identity of differentially expressed genes (DEGs) outside the intended HSD17B13 target.

### 3D MASH NAC-organoid model and pharmacological treatment

**Fig 1:**
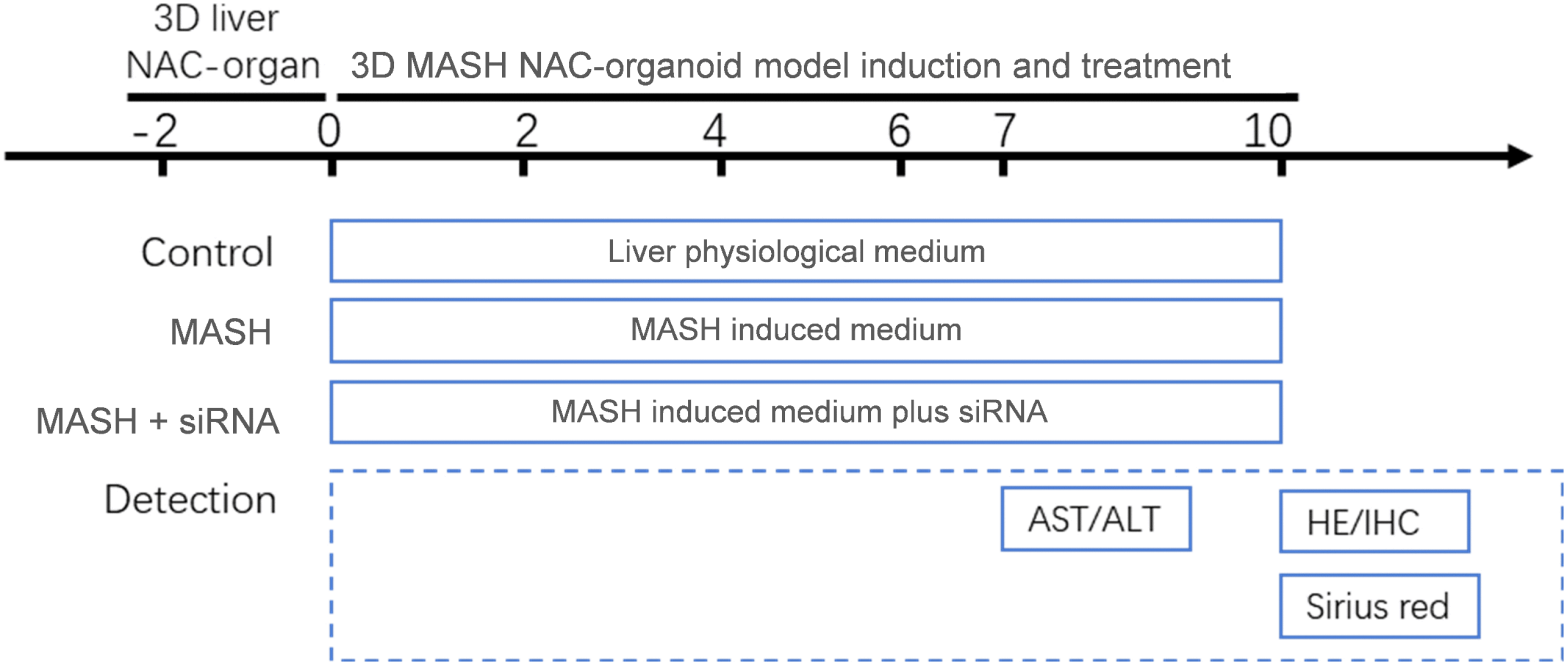
The flowchart of the 3D MASH NAC-organoid model construction. A NAC-organoid model was established within the first two days, incorporating both parenchymal and non-parenchymal cells. Hepatocytes, lSECs, KCs, and activated stellate cells were assembled based on matched NAC-linkers at a ratio of 10:6:3:1, then cultured with different medium that could induce the MASH model and physical NAC-spheroid.

The 3D MASH model was established with an NAC-Linker medium (Puheng, NAC001) and characterized by Puheng Technology (Suzhou, China) as a key human-relevant platform for efficacy testing. The organoids were formed by co-culturing primary human hepatocytes (Liver Biotechnology, LV-PHH001), primary hepatic stellate cells (Liver Biotechnology, LV-HSC001), liver sinusoidal endothelial cells (Liver Biotechnology, LV-LES001), and Kupffer cells (Liver Biotechnology, LV-Kup001) in a defined 3D culture system (Fig 1, the flow chart).

Model Induction: After a 24-hour stabilization period, the MASH phenotype was induced by replacing the maintenance medium with a MASH induction medium (Puheng, MI001) or a NAC-Liver medium (Puheng, MS01) for 10 days.

siRNA Treatment: During the 10-day induction period, organoids were treated with our lead siRNA candidates (si-R5-42, si-R5-94, si-R5-117) or the reference compound ARO-HSD at low, medium, and high concentrations. A vehicle control (PBS) and a disease control (MASH induction only) were included in every experiment.

Model Validation: Prior to efficacy studies, the model was validated to confirm the development of key MASH hallmarks, including macrovesicular steatosis (via Oil Red O staining and triglyceride (TG) content quantification), activation of hepatic stellate cells (α-SMA IHC), and collagen deposition (Sirius Red staining). Details are referred to in the reference [16].

### Histopathological and immunohistochemical analysis

Sample Processing: Designer spheroids were washed with PBS, fixed in 4% paraformaldehyde overnight at 4°C, and embedded in paraffin. Sections of 4 μm thickness were prepared for staining.

H&E and Sirius Red Staining: Hematoxylin and Eosin (H&E) staining was performed for general histological assessment and non-alcoholic fatty liver (NAFLD) Activity Score (NAS) evaluation. Sirius Red staining (Solarbio, G1340) was used to visualize and quantify collagen deposition, a key marker of fibrosis.

Immunohistochemistry (IHC): IHC for HSD17B13 protein expression was performed on a Ventana BenchMark GX automated stainer (Ventana Medical Systems) using a monoclonal anti-HSD17B13 antibody (Invitrogen, PA5-109834) and detected with the OptiView DAB IHC Detection Kit (Roche, 760-700). Staining intensity was quantified using digital image analysis with ImageJ to correlate protein knockdown with phenotypic improvement.

### Biochemical assays

TG content in organoid lysates was quantified using a commercially available enzymatic assay kit (Applygen, E1013). Levels of alanine aminotransferase (ALT) and aspartate aminotransferase (AST) in the culture supernatant were measured using chemistry kits (Juchuangbio, ALT-2070, AST-2070) to assess hepatocellular injury.

### Statistical analysis

All data are presented as mean ± standard deviation (SD). Statistical analyses were performed using GraphPad Prism 8.3. For comparisons between multiple groups, one-way or two-way ANOVA was applied, followed by post-hoc tests (Tukey’s or Dunnett’s) for multiple comparisons. A *p*-value of less than 0.05 was considered statistically significant.

## Results and discussion

### Rational design and in vitro screening identify potent HSD17B13 siRNA candidates

**Fig 2:**
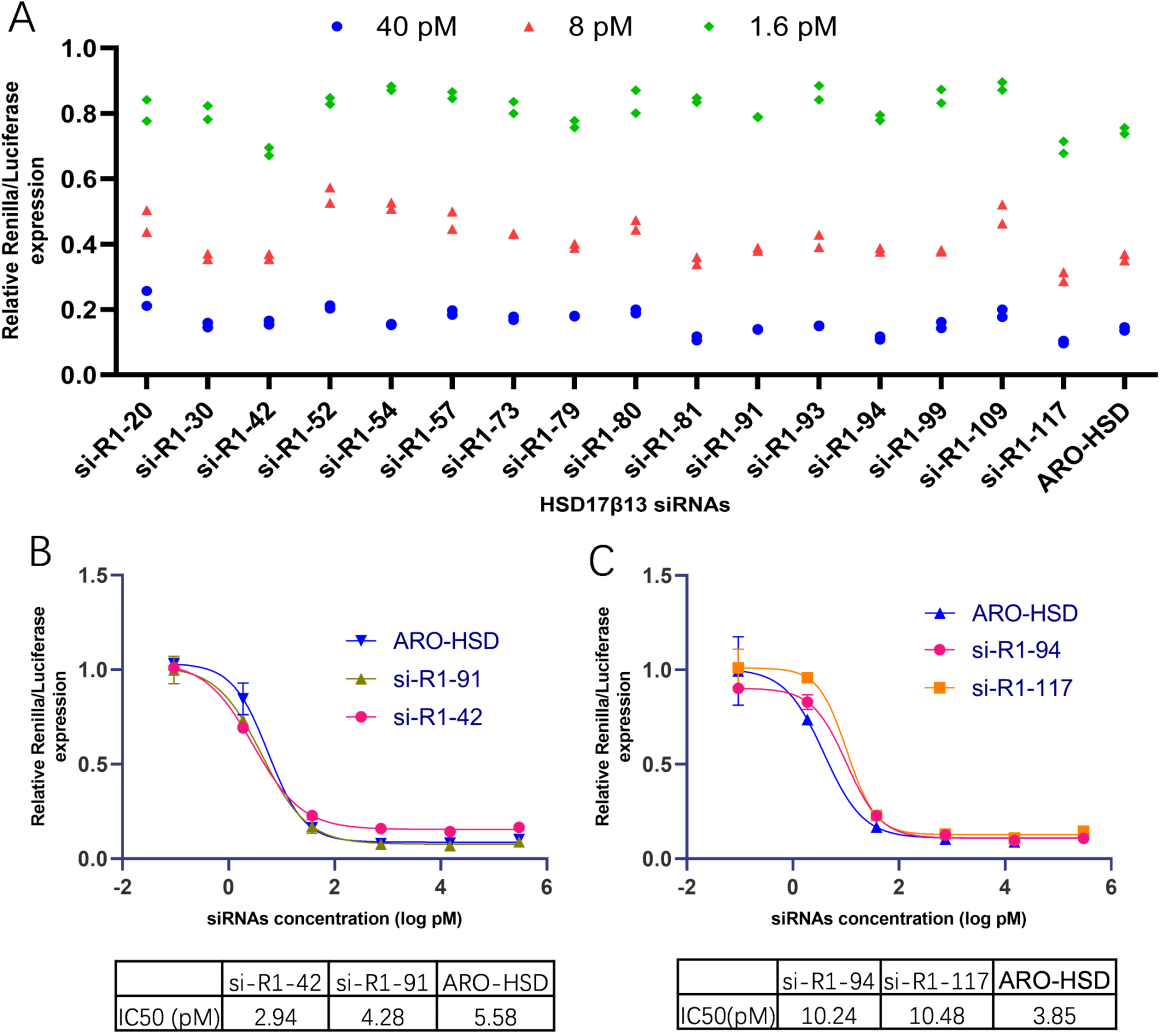
RGA results of non-modification HSD17B13 siRNAs. (A) Reduction of reporter gene assay (RGA) in the HEK293 reporter cell line by siRNA candidates. Values are plotted as a mean (s.d.) ratio of Renilla to luciferase signal. siRNA candidates were tested in duplicate at concentrations of 1.6 pM, 8 pM, and 40 pM. (B) and (C) Non-modification HSD17B13 siRNA candidates and ARO-HSD dose titration in HEK293 reporter cells. Values are plotted as a mean ± SD ratio of Renilla to luciferase signal.

We initiated our campaign by leveraging the OriSiRNA platform to design 121 unmodified siRNA candidates targeting human HSD17B13 mRNA. Primary screening using a dual-luciferase RGA in HEK293 cells identified several molecules with nanomolar to picomolar potency (Fig 2A). From these, we selected three lead sequences (si-R1-42, −91, −94, −117) based on high silencing efficacy and sequence novelty for further development. Notably, the unmodified forms of si-R1-42, −94, and −117 exhibited IC₅₀ values of approximately 10 pM and achieved greater than 80% knockdown of HSD17B13 mRNA, a level of activity comparable to the clinical benchmark, ARO-HSD (Figs 2B and 2C). Furthermore, these candidates demonstrated potent cross-reactivity with cynomolgus and rhesus monkey HSD17B13 orthologs, supporting their potential use in preclinical toxicology studies (Supplementary S1 Figure).

The initial screening successfully yielded multiple highly active siRNA sequences. The fact that our top unmodified candidates matched the potency of ARO-HSD, a molecule already demonstrating clinical efficacy, provides strong initial validation of our design platform and de-risks the subsequent optimization steps.

### Systematic chemical optimization enhances stability while maintaining potency

Unmodified siRNAs are notoriously susceptible to nuclease degradation. To address this, we embarked on a systematic, two-round chemical modification strategy on our four lead sequences. The first round (R2) introduced 2’-OMe and 2’-F modifications at various positions to identify tolerated sites [17–19]. The reporter gene activity of the first round of chemical modification was not shown. Then, the second round (R3) combined these modifications on both strands and used Huh7 reporter cells to test the silencing efficacy, as shown in Table 1.

**Table 1:** The IC50 of the second-round chemical modification HSD17β13 siRNA candidates and ARO-HSD based on RGA of the Huh7 cell line, and the relative efficacy normalized to ARO-HSD.

NO.	Huh7 cell line		NO.	Huh7 cell line	
	IC50(pM)	normalized to ARO-HSD		IC50(pM)	normalized to ARO-HSD
si-R3-42.01	58.32	18%	si-R3-91.04	9.455	240%
si-R3-42.02	62.72	23%	si-R3-91.05	4.482	206%
si-R3-42.03	75.64	19%	si-R3-91.06	11.5	80%
si-R3-42.04	71.01	20%	si-R3-94.01	61.86	14%
si-R3-42.05	39.91	36%	si-R3-94.02	77	11%
si-R3-42.06	31.29	51%	si-R3-94.03	41.06	29%
si-R3-42.07	24.64	65%	si-R3-94.04	43.5	28%
si-R3-42.08	222.2	13%	si-R3-94.05	24.07	50%
si-R3-42.09	219.2	13%	si-R3-94.06	6.783	178%
si-R3-91.01	48.54	24%	si-R3-117.01	15.02	115%
si-R3-91.02	14.06	161%	si-R3-117.02	23.83	72%
si-R3-91.03	33.51	68%	ARO-HSD	8.9 ~ 29.3	100%

**Fig 3:**
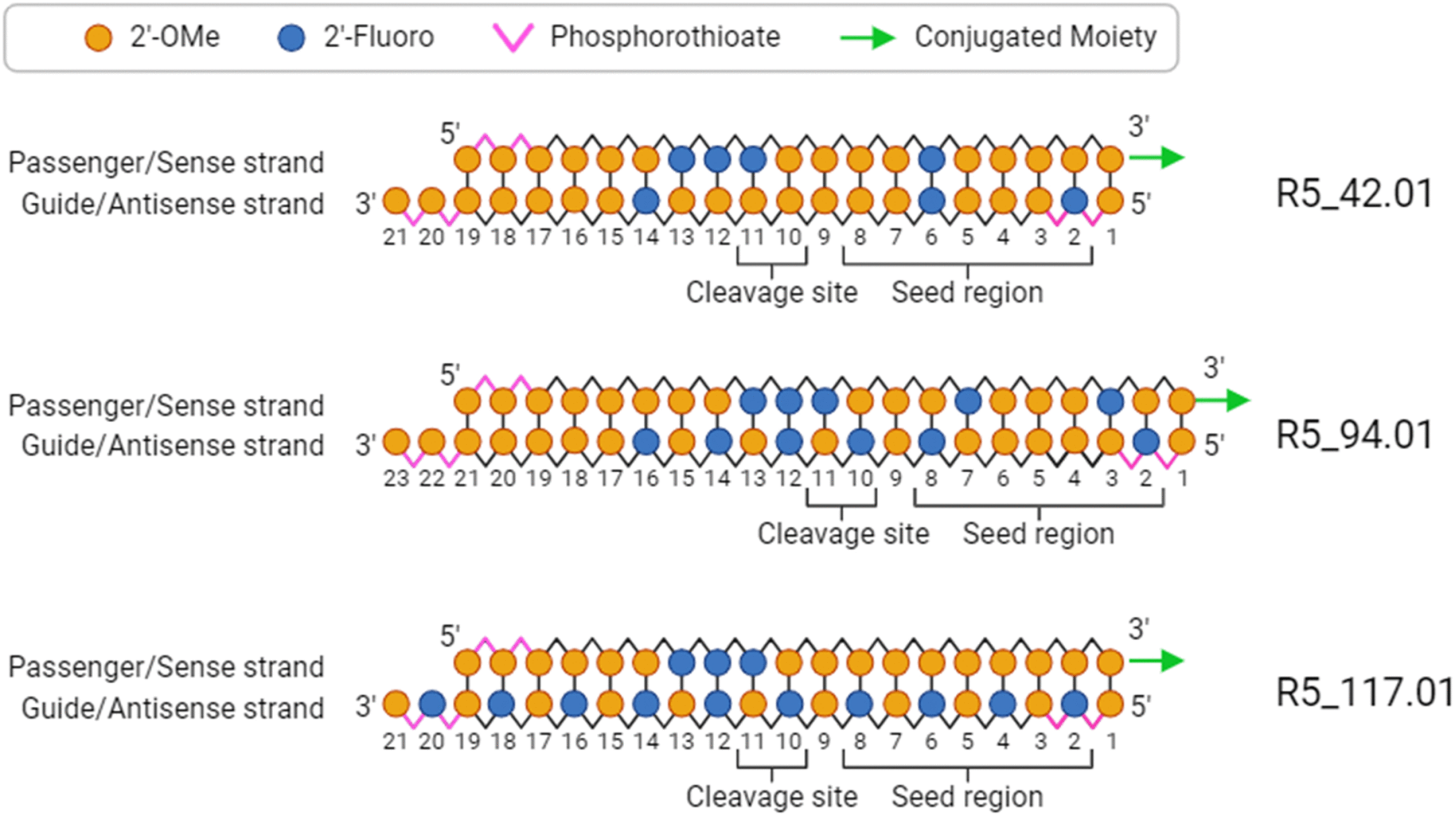
The final three candidate siRNAs and their corresponding chemical modification profiles.

Then, the leading candidates in round 3 were followed by the addition of phosphorothioate (PS) linkages (R4) to further enhance nuclease resistance [20]. The final lead candidates (si-R4-42, −94, −117) were conjugated to a GalNAc moiety at the 3’-end of the sense strand (si-R5 series, Fig 3) for targeted hepatic delivery [17,21,22].

**Fig 4:**
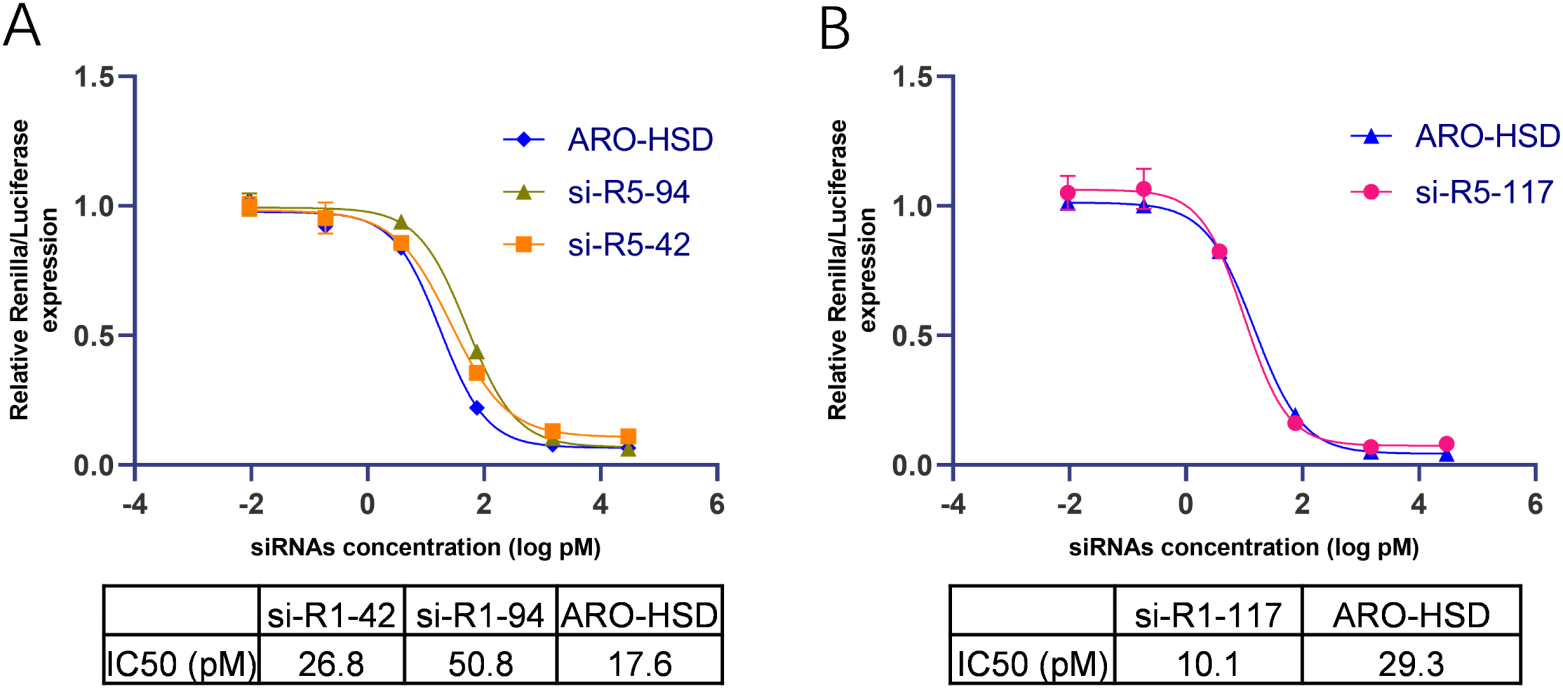
RGA results of chemical modification of HSD17B13 siRNAs. (A) and (B) Chemical modification of HSD17B13 siRNA candidates and ARO-HSD dose titration in HepG2 reporter cells. Values are plotted as the mean ± SD ratio of Renilla to luciferase signal.

**Fig 5:**
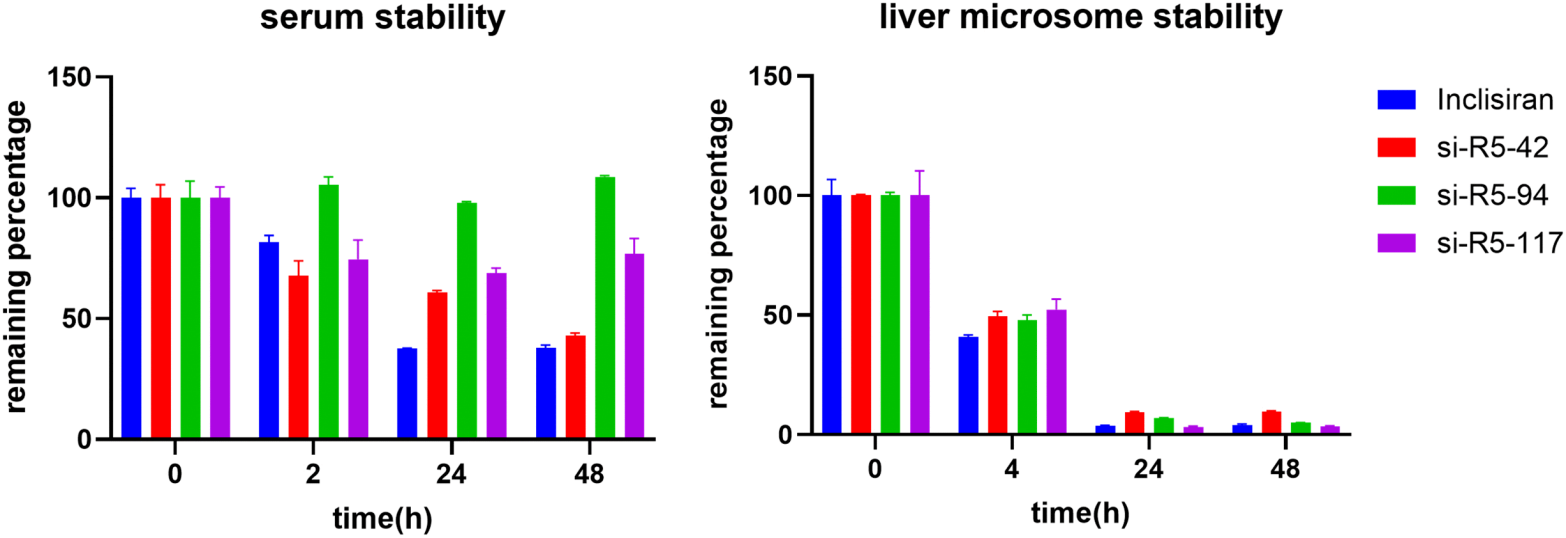
The stability was assessed at 0 hours, 2 hours, 24 hours, and 48 hours in serum and liver microsomal matrix. (A) si-R5-42, si-R5-94, and si-R5-117 were more stable than the FDA-approved siRNA drug, Inclisiran, in serum matrix. (B) si-R5-42, si-R5-94, and si-R5-117 could keep appropriate 50 percentage remaining in liver microsome and si_R5_42 was more stable than other candidates, include inclisiran. Two samples were placed at each time point, and each sample was tested twice in parallel.

Crucially, this extensive chemical engineering did not compromise biological activity. In the luciferase RGA, the final GalNAc-conjugated candidates si-R5-42, si-R5-94, and si-R5-117 maintained impressive potency, with IC₅₀ values of 26.8 pM, 50.8 pM, and 10.1 pM, respectively (Fig 4). More importantly, stability assessments in human serum and liver microsomes revealed a dramatic improvement. All three optimized siRNAs retained more than 50% of their original concentration after 48 hours in human serum, displaying stability comparable to that of Inclisiran, an FDA-approved siRNA therapeutic (Figs 5A and 5B).

The successful retention of high-potency post-modification is non-trivial, as chemical alterations can interfere with RISC loading [23,24]. Our results demonstrate a carefully balanced modification pattern that synergistically enhances metabolic stability—a critical pharmacokinetic property—without sacrificing the pharmacodynamic effect. This significantly enhances their potential for sustained target engagement in vivo.

### The prediction of off-target toxicity

RNA sequencing results were analyzed to assess the potential off-target cytotoxicity of three siRNA candidates (si-R4-42, si-R4-94, and si-R4-117). Differential expression analysis (*p* < 0.05, |log_2_FC| >= 1) confirmed that all three siRNAs significantly reduced HSD17B13 mRNA expression, demonstrating effective on-target activity. Critically, no common differentially expressed genes (DEGs) were identified across all three candidates, indicating the absence of a shared off-target mRNA silencing effect. Furthermore, each siRNA exhibited minimal unique off-target DEGs, collectively suggesting a low off-target cytotoxicity profile for these candidates.

### Efficacy validation in a human 3D MASH organoid model

Given the known species-specific limitations of murine MASH models [14,16], we employed a biologically complex human 3D NAC-organoid model to evaluate therapeutic efficacy. This model recapitulates key disease hallmarks, including steatosis, inflammation, and fibrosis, providing a human-relevant system for preclinical validation [16]. After 10 days of treatment, several key MASH-related parameters were evaluated.

#### Amelioration of steatosis and histological severity.

**Fig 6:**
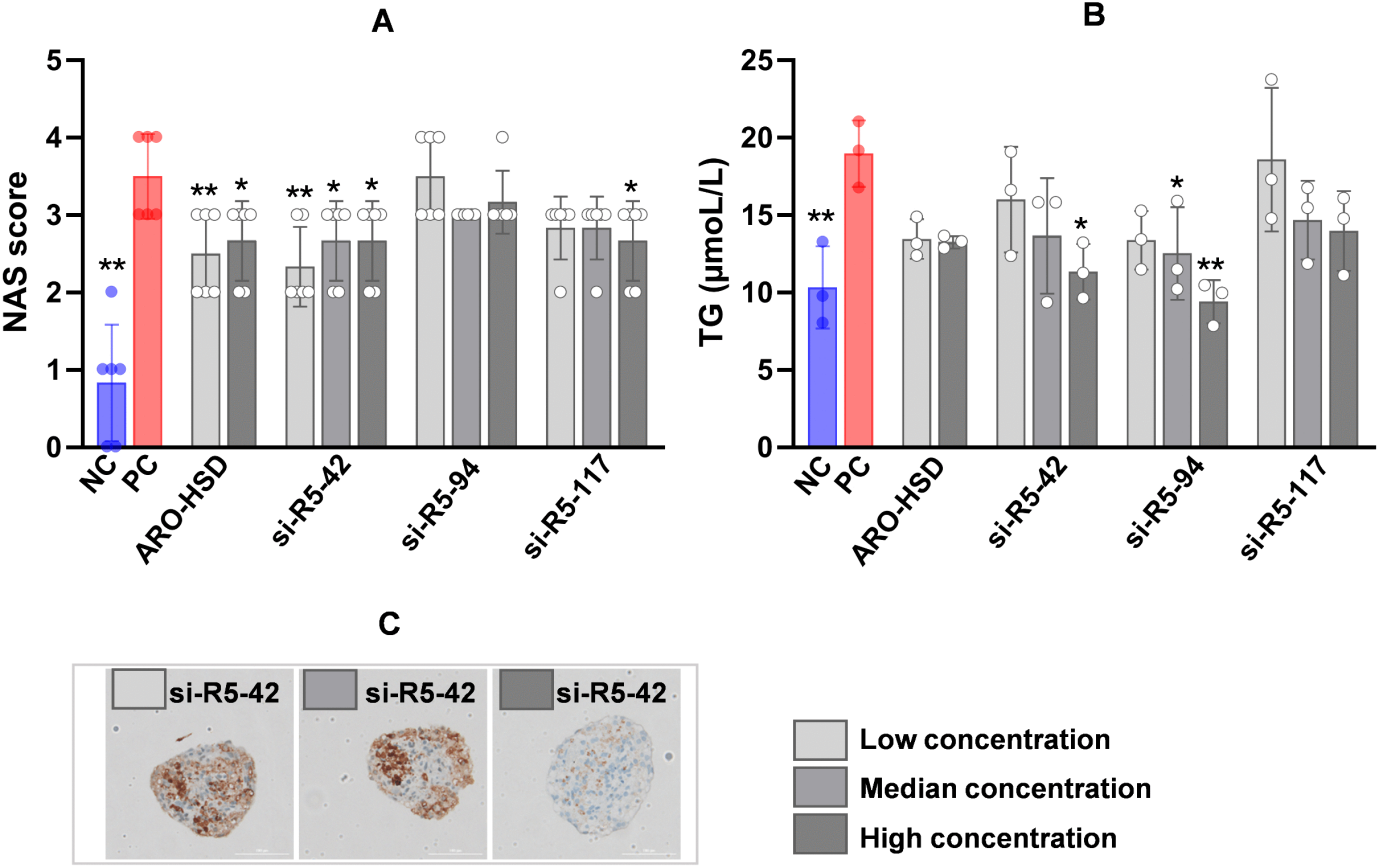
The results of NAS, TG, and HSD17B13 protein expression in the 3D MASH NAC-Organ model. (A) Results from the NAS score, every group had 1–2 score improvements. NAS scores were assessed by two pathology scientists. (B) si-R5-42, si-R5-94, and si-R5-117 showed a dose-dependent improvement in TG accumulation, of which si-R5-42 and si-R5-94 performed better. (C) The expression of HSD17B13 protein in cells treated with si-R5-42 at low, medium, and high concentrations was detected by IHC, and the results showed that si-R5-42 reduced HSD17B13 protein expression in a dose-dependent manner. Each group was tested at concentrations of 2 nM, 10 nM, and 50 nM. Data are mean ± SD of n = 3. *P < 0.05 and **P < 0.01. NC, Normal physiological control with essential medium. PC, Positive pathological control with MASH-induced medium.

**Fig 7:**
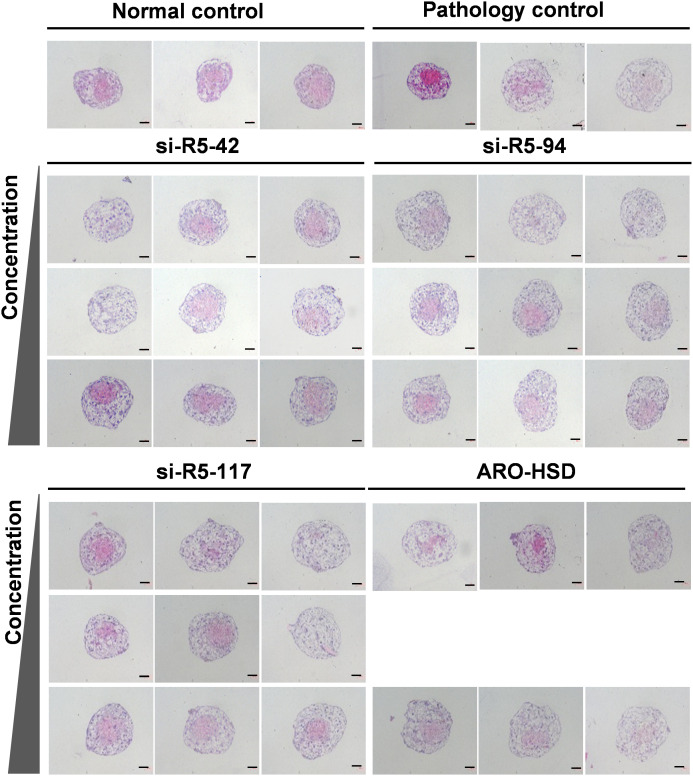
The results of fibrosis assessment in the 3D MASH NAC-Organ model. si-R5-42, si-R5-94, and si-R5-117 showed a dose-dependent improvement in fibrosis accumulation, of which si-R5-42 performed better. Each group was tested at concentrations of 2 nM, 10 nM, and 50 nM. Data are mean ± SD of n = 3. *P < 0.05 and **P < 0.01. NC, Normal physiological control with essential medium. PC, Positive pathological control with MASH-induced medium.

Treatment with our lead siRNAs during MASH induction resulted in a significant and dose-dependent improvement in liver pathology. Histological analysis (H&E staining) showed a marked reduction in the NAFLD Activity Score (NAS) across all treatment groups compared to the disease control (Figs 6A and 7).

This histological improvement was corroborated by a direct measurement of triglyceride (TG) content. si-R5-42 treatment produced a dose-dependent reduction in TG accumulation, the magnitude of which exceeded that achieved by ARO-HSD at a comparable dose (Fig 6B). Immunohistochemistry confirmed that this robust phenotypic response was correlated with a profound knockdown of HSD17B13 protein expression (Fig 6C). Notably, at high doses, our candidates, si-R5-42, elicited a more pronounced reduction in TG than ARO-HSD, highlighting their potential to downregulate TG in this human system.

The superior performance of our lead candidate, si-R5-42, over ARO-HSD in reducing TG in a human model is a pivotal finding. It suggests that our integrated optimization strategy may have yielded a molecule with enhanced therapeutic potential. This outcome also highlights the importance of using human-relevant models for candidate selection, as such nuances may be overlooked in conventional animal studies.

#### Attenuation of fibrogenesis.

**Fig 8:**
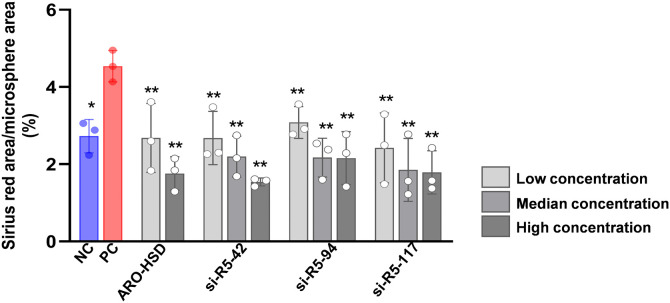
Hematoxylin and eosin staining in the 3D MASH NAC-Organ model. Each group was tested in triplicate at concentrations of 2 nM, 10 nM, and 50 nM.

**Fig 9:**
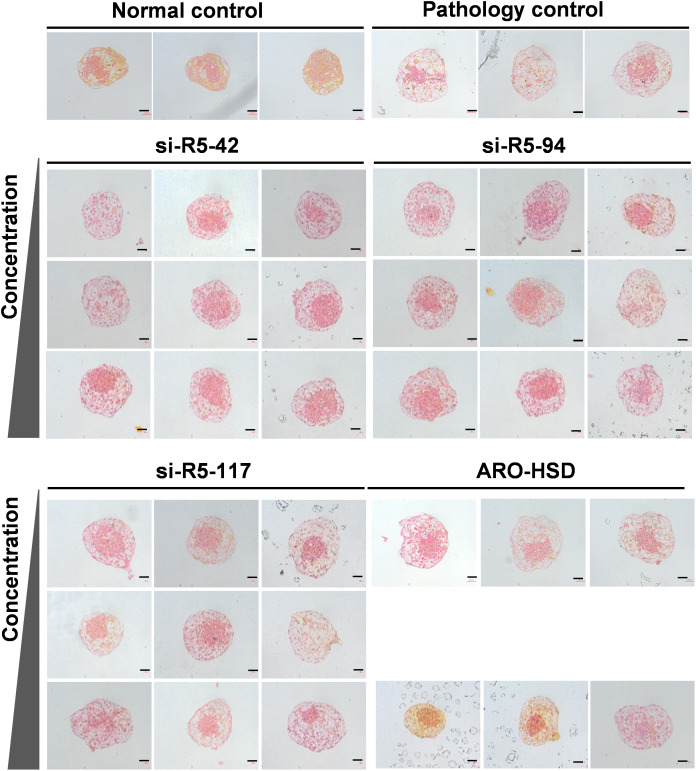
Sirius red staining in the 3D MASH NAC-Organ model. Each group was tested in triplicate at concentrations of 2 nM, 10 nM, and 50 nM.

A critical challenge in MASH treatment is halting or reversing the progression of fibrosis. Sirius Red staining revealed that silencing HSD17B13 expression with our candidates resulted in a significant reduction in collagen deposition compared to the disease control (Figs 8 and 9). This demonstrates that HSD17B13 knockdown not only improves steatosis but also directly attenuates the pro-fibrotic pathway in a multicellular human liver context. These findings are consistent with seminal human genetic studies, which report that a loss-of-function (LOF) variant in HSD17B13 (rs72613567) is associated with a significantly reduced risk of progression from steatosis to advanced fibrosis, cirrhosis, and hepatocellular carcinoma. Our data functionally validate this protective association by demonstrating that pharmacological silencing of HSD17B13 recapitulates the anti-fibrotic effect observed in human carriers of the LOF variant [25]. The ability of our model to detect anti-fibrotic efficacy positions it as a superior, human-relevant tool for validating the therapeutic potential of drugs targeting this critical pathological process.

#### Analysis of transaminase levels.

Analysis of culture supernatants revealed that ALT and AST levels in the disease control were paradoxically lower than in the healthy control (Supplementary S1 Table). We hypothesize that this may be due to the dynamic injury-repair processes within the organoids or technical limitations in sensitivity for small-volume samples. Despite this, the clear and consistent improvements in histology (the gold standard for MASH assessment) and fibrosis provide compelling evidence of the therapeutic benefit of HSD17B13 silencing.

## Conclusion

Our study successfully establishes an integrated and translational pipeline for the development of siRNA therapeutics, moving from rational in silico design to functional validation in a human-relevant system. While we confirmed the inherent promise of siRNA for treating chronic metabolic diseases like MASH, our work specifically addresses the critical pitfalls of stability and, most importantly, the translational gap between preclinical models and clinical outcomes.

The cornerstone of this strategy was the deployment of a sophisticated human 3D NAC-organoid model of MASH. This model proved indispensable, as it recapitulated the multicellular complexity and key disease hallmarks of human MASH, providing a platform with superior predictive power over conventional animal models. It was within this clinically relevant context that we could rigorously benchmark our lead candidate, si-R5-42.

Our data compellingly demonstrate that si-R5-42 is not merely a research tool but a robust therapeutic candidate. It combines picomolar silencing potency with greatly enhanced stability in human serum and liver microsomes, and a low risk of off-target effects in vitro. Crucially, in the 3D organoid model, si-R5-42 elicited a significant therapeutic response, reducing hepatic steatosis, attenuating fibrogenesis, and improving overall histology. The fact that si-R5-42 achieved this with efficacy comparable to the clinical-phase candidate ARO-HSD.

In conclusion, this research delivers a dual advancement. First, we present si-R5-42 as a highly optimized, promising candidate for the treatment of MASH. Second, and more broadly, we validate a new paradigm for preclinical siRNA development. By prioritizing human-pathophysiologically relevant models over species-divergent animal systems for critical efficacy studies, we have established a generalizable pipeline that can accelerate and improve the success rate of future RNAi therapeutics, not only for liver diseases but for a wide range of complex human conditions.

## Supporting information

Fig S1 Cross-reaction study in rhesus monkey and cynomolgus monkey. si-R1-42, si-R1-91, si-R1-94, si-R1-117 and ARO-HSD dose titration in HEK293-psiCHECK-rhesusHSD17B13 or in HEK293-psiCHECK-cynoHSD17B13 reporter cells. Mean±SD reporter gene expression versus log-pmol siRNA concentration, n = 3.
(TIF)

Table S1 ALT and AST concentration results, mean±SD, n = 3. (DOCX)
