# Supplementary material for: A chemically optimized, GalNAc-conjugated siRNA targeting HSD17B13 demonstrates efficacy in a human 3D organoid model of MASH, showcasing an alternative to animal testing
Source: PLoS One. 2026 Aug 14;21(8):e0354751. doi: 10.1371/journal.pone.0354751 (PMC13475973; doi:10.1371/journal.pone.0354751)
Supplement: Supplementary file 1 [file pone.0354751.s002.docx]

| **Group** | **ALT (U/L)** | **AST (U/L)** |
| --- | --- | --- |
| Normal physiological control | 1.37 ± 0.68 | 3.07 ± 0.90 |
| Positive pathological control | 1.33 ± 0.69 | 6.27 ± 1.51 |
| ARO-HSD-low | 3.03 ± 0.97 | 5.83 ± 0.82 |
| ARO-HSD-high | 2.90 ± 0.70 | 7.77 ± 1.55 |
| si-R5-42-low | 2.40 ± 0.94 | 5.63 ± 1.60 |
| si-R5-42-median | 1.73 ± 0.21 | 6.96 ± 0.48 |
| si-R5-42-high | 1.83 ± 0.78 | 4.13 ± 0.42 |
| si-R5-94-low | 1.87 ± 0.38 | 7.73 ± 1.03 |
| si-R5-94-median | 2.70 ± 1.10 | 6.40 ± 1.42 |
| si-R5-94-high | 2.27 ± 0.76 | 6.70 ± 1.36 |
| si-R5-117-low | 3.73 ± 1.38 | 8.00 ± 0.43 |
| si-R5-117-median | 3.63 ± 0.65 | 6.80 ± 0.93 |
| si-R5-117-high | 4.50 ± 1.76 | 6.87 ± 2.04 |

Supplementary Table S1 ALT and AST concentration results, mean±SD, n=3.
